# Glycogen storage disease presenting as Cushing syndrome

**DOI:** 10.1002/jmd2.12031

**Published:** 2019-04-03

**Authors:** Margaret A. Stefater, Joseph I. Wolfsdorf, Nina S. Ma, Joseph A. Majzoub

**Affiliations:** ^1^ Division of Endocrinology, Department of Pediatrics Boston Children's Hospital, Harvard Medical School Boston Massachusetts

**Keywords:** cortisol, glycogen storage disease, growth, hypothalamic‐pituitary‐adrenal axis, pseudo‐Cushing syndrome, stress‐induced Cushing (SIC) syndrome

## Abstract

Impaired growth is common in patients with glycogen storage disease (GSD), who also may have “cherubic” facies similar to the “moon” facies of Cushing syndrome (CS). An infant presented with moon facies, growth failure, and obesity. Laboratory evaluation of the hypothalamic‐pituitary‐adrenal (HPA) axis was consistent with CS. He was subsequently found to have liver disease, hypoglycemia, and a pathogenic variant in *PHKA2*, leading to the diagnosis of GSD type IXa. The cushingoid appearance, poor linear growth and hypercortisolemia improved after treatment to prevent recurrent hypoglycemia. We suspect this child's HPA axis activation was “appropriate” and caused by chronic hypoglycemic stress, leading to increased glucocorticoid secretion that may have contributed to his poor growth and excessive weight gain. This is in contrast to typical CS, which is due to excessive adrenocorticotropic hormone (ACTH) or cortisol secretion from neoplastic pituitary or adrenal glands, ectopic secretion of ACTH or corticotropin‐releasing hormone (CRH), or exogenous administration of corticosteroid or ACTH. Pseudo‐CS is a third cause of excessive glucocorticoid secretion, has no HPA axis pathology, is most often associated with underlying psychiatric disorders or obesity in children and, by itself, is thought to be benign. We speculate that some diseases, including chronic hypoglycemic disorders such as the GSDs, may have biochemical features and pathologic consequences of CS. We propose that excessive glucocorticoid secretion due to chronic stress be termed “stress‐induced Cushing (SIC) syndrome” to distinguish it from the other causes of CS and pseudo‐CS, and that evaluation of children with chronic hypoglycemia and poor statural growth include evaluation for CS.

## 1 INTRODUCTION

Children with undiagnosed hepatic glycogen storage disease (GSD) are classically characterized by “cherubic” facies and impaired growth.^1^, ^2^, ^3^ Short stature in patients with GSD has been correlated with plasma cortisol levels^1^, ^3^ but potential overlap with Cushing syndrome (CS) has not been investigated.

Typically, CS is due to abnormally increased hormone secretion from neoplastic pituitary (ACTH) or adrenal (cortisol) glands, ectopic secretion of ACTH and/or CRH, or administration of excessive corticosteroid or ACTH.^4^ It is treated by surgical removal of the source of abnormal hormone secretion or cessation of the exogenous drug. Pediatric CS is characterized by poor linear growth associated with preserved or augmented weight gain.^4^, ^5^ It is rare in children and is among the most challenging endocrine diseases to diagnose because its symptoms and signs can overlap with other, more common, medical disorders^5^ including obesity and depression.^6^, ^7^, ^8^ The presence of elevated cortisol secretion in these other conditions has been termed “pseudo‐Cushing” syndrome to distinguish them from classical CS that requires further evaluation and treatment.^9^, ^10^, ^11^, ^12^, ^13^ Diagnostic testing for CS is technically challenging in young children. Batista et al^14^ recommend inpatient testing, including measurement of midnight serum cortisol levels and 24‐hour urinary free cortisol (UFC) excretion as highly sensitive and specific markers for pediatric CS.^6^ However, because the biochemical profile is similar in pseudo‐CS, it may be difficult to distinguish between the two. In obese children, normal growth velocity favors pseudo‐CS over CS.^6^

Here, we describe an infant who was initially evaluated for CS because of moon facies, growth failure, obesity and hypercortisolemia, and was subsequently diagnosed with GSD IXa. The cushingoid appearance, poor linear growth and hypercortisolemia improved after instituting treatment to prevent recurrent hypoglycemia. We suspect that this child's chronic hypoglycemia led to hypothalamic‐pituitary‐adrenal (HPA) axis activation, which contributed to his initial growth failure and weight gain, both of which were reversed upon amelioration of the hypoglycemia. We speculate that some diseases associated with chronic hypoglycemia, such as the GSDs, may be associated with activation of the HPA axis. Although this activation is a physiologically appropriate counter‐regulatory response, it may nevertheless have pathologic consequences that contribute to the GSD phenotype of growth failure and obesity, leading us to propose it as a novel type of CS, which we term “stress‐induced Cushing (SIC) syndrome”.

## 2 CASE REPORT

**Figure 1:**
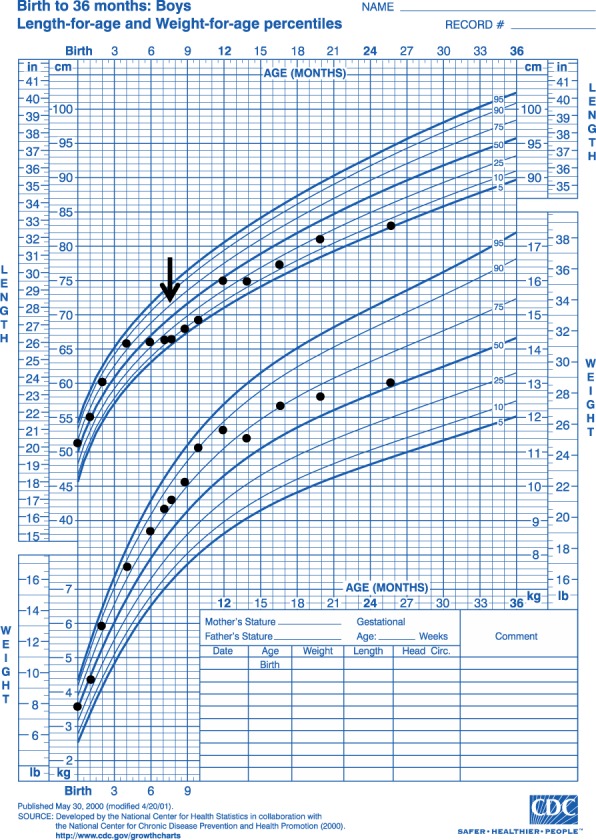
CDC growth chart. Arrow indicates time of diagnosis and initiation of dietary therapy

**Figure 2:**
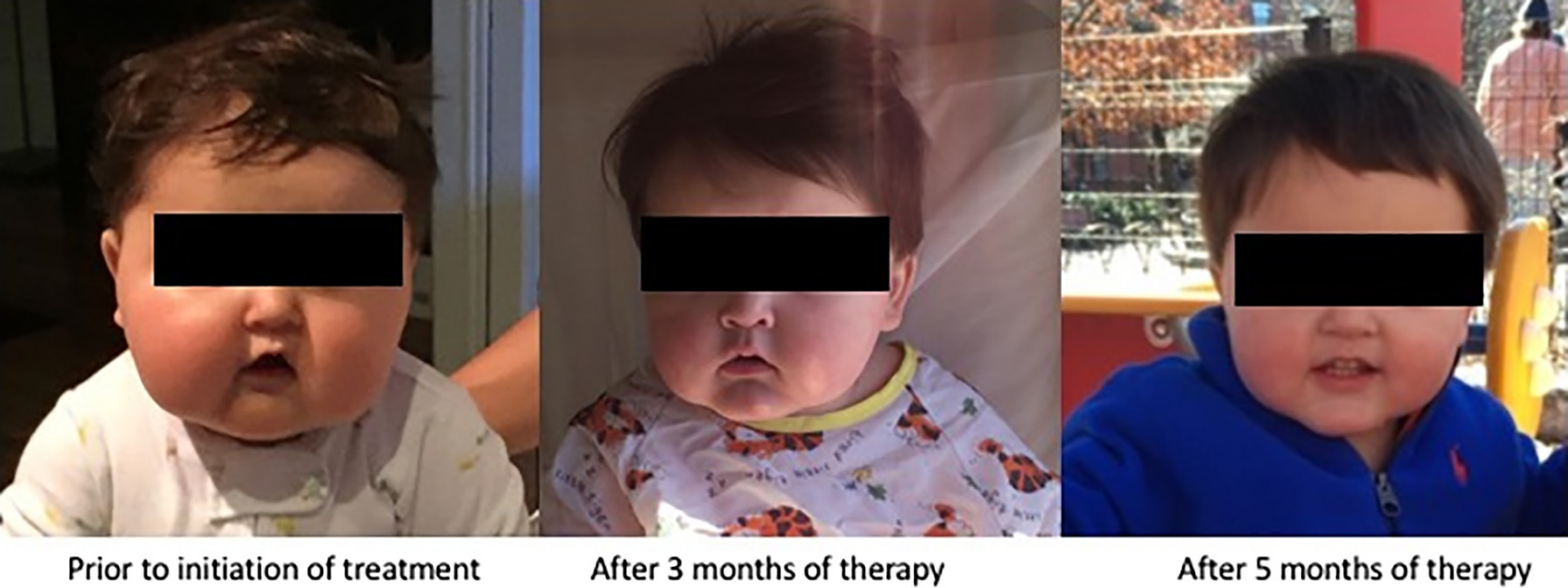
Facial appearance of patient

A 7‐month‐old male was referred to our hospital due to poor linear growth and excessive weight gain (Figure 1). He was fed a combination of breast milk and formula and had begun to sleep through the night a few months before presentation. He was the product of a healthy, full‐term pregnancy; newborn screening tests were normal. There was no prior history of hypoglycemia or symptoms suggestive of hypoglycemia, seizures or midline cranial defects. His neurodevelopment was appropriate for age. He had not received corticosteroids. His growth chart showed a marked decrease in length percentiles from 73rd to 5th between ages 4 and 7 months, with a length‐for‐age *z*‐score of −1.66 SD at the time of our evaluation. On physical examination, he was obese and had plethoric moon facies (Figure 2); abdominal examination was difficult and could not exclude organomegaly or an abdominal mass.

An evaluation for CS was performed (Table 1). Initial laboratory studies included serum cortisol and plasma ACTH in the afternoon, which were 14.4 mcg/dL and 12 pg/mL, respectively. Thyroid function tests were normal and, although insulin‐like growth factor 1 (IGF‐1) was low, insulin‐like growth factor binding protein‐3 (IGFBP‐3) was normal. Diurnal assessment of adrenal function revealed that midnight (2400 hours) serum cortisol concentration was high on two occasions (10.4 and 7 mcg/dL), and despite appropriately high serum dexamethasone, administration of low‐dose dexamethasone did not suppress his morning serum cortisol (9.8 mcg/dL). Two twenty‐four hour UFC measurements were not elevated, but these data were probably unreliable owing to observed leakage around the bladder catheter and low urine creatinine excretion, suggesting incomplete collection.

**Table 1:** Laboratory data

Serum studies	Baseline^a^	Diurnal				Suppression^b^		Normal
		Day 1 08:30	Day 1 24:00	Day 2 06:00	Day 2 24:00	ON DST 1	ON DST 2	
Free T4 (ng/dL)	1.29							0.80‐1.80
TSH (uIU/mL)	4.07							0.80‐8.20
IGF‐1 (ng/mL)	<25.0							63‐279
IGFBP‐3 (mcg/mL)	2.4							1.4‐5.2
Cortisol (mcg/dL)	14.4	20.4	10.4	18.4	7.0	9.8	1.8	5‐25
ACTH (pg/mL)	12	7	9	10	6	NA	6	≤46
Dexamethasone level (mcg/dL)						NA	89.3	NA
*Urine studies*	*Collection 1*			*Collection 2*				*Normal*
24‐hour UFC	5.7 mcg			3.9 mcg				3‐9 mcg
24‐hour urine creatinine	76 mg (7.75 mg/kg)			33 mg (3.36 mg/kg)				~10 mg/kg/day^22^
Urine volume	475 mL			550 mL				NA

a Collected at 17:33 hours.

b Low‐dose (0.015 mg/kg) overnight dexamethasone suppression test (ON DST) 2 was performed after euglycemia was achieved.

A pituitary magnetic resonance imaging (MRI) study was normal. Abdominal ultrasound did not show adrenal pathology, but incidentally revealed hepatomegaly. Further diagnostic evaluation showed markedly elevated transaminase levels (AST 2001, ALT 921 U/L) and hypertriglyceridemia (779 mg/dL) as well as recurrent fasting hypoglycemia with plasma glucose levels as low as 21 mg/dL. Molecular genetic testing identified a hemizygous, known pathogenic mutation in *PHKA2* (c.3505C>T), confirming the diagnosis of hepatic phosphorylase kinase deficiency (Type IXa GSD).^15^ The patient's hypoglycemia resolved after the introduction of a regimen of frequent carbohydrate‐ and protein‐enriched feedings. Once a period of stable euglycemia (blood glucose levels ≥70 mg/dL) had been achieved, an overnight low‐dose dexamethasone suppression test was repeated, which showed normal suppression of the morning serum cortisol to 1.8 mcg/dL (Table 1). This suggested resolution of a hypercortisolemic state that was secondary to unrecognized recurrent hypoglycemia. Following the initiation of dietary therapy, the patient's statural growth improved, and his weight fell from the 76th to the 50th percentile (Figure 1), and his cushingoid appearance improved (Figure 2).

## 3 DISCUSSION

We describe an infant who presented with growth failure, abnormal weight gain, moon facies, and hypercortisolism, who was ultimately diagnosed with Type IXa GSD. Signs and laboratory evidence of CS reversed following treatment for, and resolution of, hypoglycemia. We hypothesize that unrecognized hypoglycemia led to chronic HPA axis activation in our patient, and that this led to the patient's hypercortisolism and its associated manifestations.

Hypoglycemia activates counter‐regulatory mechanisms leading to increased secretion of glucagon, epinephrine, growth hormone (GH), and cortisol.^16^ Patients with GSD can mount a normal counter‐regulatory hormone response to hypoglycemia, but this is futile due to an enzymatic defect in hepatic glycogenolysis.^17^ We propose that in our patient, persistent and recurrent hypoglycemia led to chronic HPA axis activation, causing poor linear growth and obesity. His striking physical appearance and growth pattern initially led to a presumptive diagnosis of CS.^4^, ^18^ Midnight serum cortisol levels were ≥7.0 mcg/dL on two separate occasions, and endogenous cortisol production failed to suppress after low‐dose overnight dexamethasone administration. Midnight serum cortisol levels have been shown to be highly sensitive and specific for CS; a single midnight cortisol level ≥ 4.4 mcg/dL has been shown in children to have 99% sensitivity and 100% specificity.^14^ After diagnosis and treatment of GSD IXa, stable overnight euglycemia was achieved, and a morning serum cortisol concentration suppressed normally in response to low‐dose dexamethasone. The resolution of both biochemical hypercortisolism and growth failure after restoring euglycemia strongly suggests a diagnosis of secondary, reversible CS in our patient.

The patient exhibited profound, early growth failure with excess weight gain, which are the clinical hallmarks of pediatric CS.^4^, ^18^ We suggest that our patient's growth deceleration was due to stressor‐induced Cushing syndrome. Although his IGF‐1 level was low, we do not believe he had primary growth hormone deficiency, since his IGFBP‐3 was normal, he did not have other hallmarks of primary GH deficiency such as microphallus, midline cranial defects, or structural abnormalities of the pituitary gland, and his growth rate improved after initiation of treatment for GSD Type IXa and resolution of hypoglycemia.

Poorly controlled GSD with frequent hypoglycemia is associated with growth failure,^3^, ^19^, ^20^ and although excess weight gain in patients with GSD has been attributed to overfeeding, there are sparse data available regarding pretreatment weight trajectories. Growth failure in GSD correlates directly with serum cortisol levels,^1^, ^3^ supporting the hypothesis that it may be due, at least in part, to hypercortisolism. Growth has been shown to improve in children with GSD 1 who undergo intensive treatment,^2^, ^19^ perhaps reflecting HPA axis normalization in response to restoration of euglycemia. Interestingly, children with GSD IX exhibit catchup growth in adolescence despite lack of specific treatment,^2^, ^21^ but this may reflect the mild nature of the disease in many patients and the fact that glucose requirements decline with age.

Our case highlights an association between chronic, intermittent hypoglycemia and features of CS. We propose that the elevated cortisol secretion in this patient with undiagnosed GSD might have been due to a previously unrecognized type of CS not caused by pathology of the pituitary or adrenal glands or exogenous corticosteroid intake. This new category of CS is differentiated from pseudo‐CS by having detrimental functional consequences (ie, growth failure and obesity), whereas the latter is often considered benign. We speculate that this type of CS could also be relevant to other diseases that may exhibit chronic activation of the HPA axis. In these situations, the term ‘pseudo’ (Greek ψευδής, pseudes, false) is not appropriate, since that should be reserved for patients with elevated cortisol without pathologic consequences. To avoid confusion with pseudo‐CS, we propose that CS due to stressor‐induced activation of the HPA axis, such as hypoglycemia in the setting of GSD, be termed “stress‐induced Cushing (SIC) syndrome.” We suggest that in children presenting with CS, chronic hypoglycemia be considered in the differential diagnosis and, conversely, that evaluation of patients presenting with chronic hypoglycemia and poor statural growth include assessment for CS. In addition, recognition of SIC as a contributor to growth failure in chronic disease states may lead to the development of novel therapeutics, including glucocorticoid antagonists, to address these complications of hypercortisolism in diseases in which the underlying pathology is not readily treatable.

## AUTHOR CONTRIBUTIONS

MS and NM directed the patient's initial care and diagnosis, with input from JM and JW. JM conceived of the concept that we then termed “stress‐induced CS.” MS wrote the manuscript, and all authors participated in discussing and editing the manuscript.

## CONFLICT OF INTEREST

No authors disclose any competing interests.

## ETHICS APPROVAL

This case report was deemed by the Boston Children's Hospital IRB to be exempt from human subjects research.

## PATIENT CONSENT

The patient's parents have consented to the use of de‐identified clinical information and photos to be used for this case report.
